# Neurohormonal activation induces intracellular iron deficiency and mitochondrial dysfunction in cardiac cells

**DOI:** 10.1186/s13578-021-00605-5

**Published:** 2021-05-17

**Authors:** M. Tajes, C. Díez-López, C. Enjuanes, P. Moliner, J. L. Ferreiro, A. Garay, S. Jiménez-Marrero, S. Yun, S. G. Sosa, L. Alcoberro, J. González-Costello, E. García-Romero, L. Yañez-Bisbe, B. Benito, J. Comín-Colet

**Affiliations:** 1 Bio-Heart Cardiovascular Diseases Research Group, Bellvitge Biomedical Research Institute (IDIBELL), L’Hospitalet de Llobregat, Barcelona, Spain; 2 Cardiology Department, Bellvitge University Hospital, L’Hospitalet de Llobregat, Barcelona, Spain; 3 Advanced Heart Failure and Heart Transplant Unit, Cardiology Department, Bellvitge University Hospital, L’Hospitalet de Llobregat, Barcelona, Spain; 4 Community Heart Failure Program, Cardiology Department, Bellvitge University Hospital, L’Hospitalet de Llobregat, Barcelona, Spain; 5 Vascular Biology and Metabolism Program, Vall d’Hebron Research Institute (VHIR), Barcelona, Spain; 6 Cardiology Department, Hospital Vall d’Hebron Hospital, Barcelona, Spain; 7 Department of Clinical Sciences, School of Medicine, University of Barcelona, Barcelona, Spain; 8 Department of Medicine, Universitat Autònoma de Barcelona, Barcelona, Spain; 9 Department of Internal Medicine, Bellvitge University Hospital, L’Hospitalet de Llobregat, Barcelona, Spain

**Keywords:** Neurohormonal activation, Heart failure, Iron deficiency, Cardiac cell, Mitochondria function

## Abstract

**Background:**

Iron deficiency (ID) is common in patients with heart failure (HF) and is associated with poor outcomes, yet its role in the pathophysiology of HF is not well-defined. We sought to determine the consequences of HF neurohormonal activation in iron homeostasis and mitochondrial function in cardiac cells.

**Methods:**

HF was induced in C57BL/6 mice by using isoproterenol osmotic pumps and embryonic rat heart-derived H9c2 cells were subsequently challenged with Angiotensin II and/or Norepinephrine. The expression of several genes and proteins related to intracellular iron metabolism were assessed by Real time-PCR and immunoblotting, respectively. The intracellular iron levels were also determined. Mitochondrial function was analyzed by studying the mitochondrial membrane potential, the accumulation of radical oxygen species (ROS) and the adenosine triphosphate (ATP) production.

**Results:**

Hearts from isoproterenol-stimulated mice showed a decreased in both mRNA and protein levels of iron regulatory proteins, transferrin receptor 1, ferroportin 1 and hepcidin compared to control mice. Furthermore, mitoferrin 2 and mitochondrial ferritin were also downregulated in the hearts from HF mice. Similar data regarding these key iron regulatory molecules were found in the H9c2 cells challenged with neurohormonal stimuli. Accordingly, a depletion of intracellular iron levels was found in the stimulated cells compared to non-stimulated cells, as well as in the hearts from the isoproterenol-induced HF mice. Finally, neurohormonal activation impaired mitochondrial function as indicated by the accumulation of ROS, the impaired mitochondrial membrane potential and the decrease in the ATP levels in the cardiac cells.

**Conclusions:**

HF characteristic neurohormonal activation induced changes in the regulation of key molecules involved in iron homeostasis, reduced intracellular iron levels and impaired mitochondrial function. The current results suggest that iron could be involved in the pathophysiology of HF.

## Background

Heart failure (HF) is a devastating condition and represents a challenge for public healthcare systems [1]. The current understanding of its pathophysiology is based on the “neurohormonal hypothesis”, which states that HF progression is promoted by the long-term maladaptive and deleterious effects of sustained neurohormonal activation in the heart and in rest of the cardiovascular system [2, 3]. Many of classical neurohormones such as norepinephrine (Nor) and angiotensin II (Ang II) are known to be synthesized directly within the myocardium and, therefore, act in an autocrine or paracrine manner [3, 4]. In fact, the sustained activation of these pathways lead to an increased plasma Ang II, epinephrine and Nor levels [5, 6]. The inhibition of these neurohormonal systems has demonstrated a consistent reduction in morbidity and mortality in patients with systolic HF and is the basis of modern pharmacological treatment [7, 8].

However, these effective therapies have failed to promote a complete remission of symptoms and restore life expectancy in many patients. This has motivated an increasing interest in emerging therapeutic targets. In this regard, iron deficiency (ID) is present in up to 50% of HF patients [9, 10] and is associated with a higher risk of mortality and hospitalization [10, 11], reduced functional capacity, and impaired health-related quality of life [12], independent of the presence of anemia [10, 12, 13]. Interestingly, intravenous iron supplementation has been recommended by European Society of Cardiology (ESC) HF guidelines as a potential therapeutic approach, since the correction of ID with intravenous iron alleviates HF symptoms, reduces risk of hospitalization and improves quality of life [14–16].

Although ID has been mainly considered as an extra-cardiac co-morbidity complicating the course of HF, recent data suggest that it may actually interact directly with the mechanisms involved in HF initiation and progression [17–20]. Previous studies from our group suggested an association between HF neurohormonal activation and systemic ID [17]. It has been shown that in chronic HF patients, the increase in the sympathetic activity (measured by Nor systemic levels) was associated with ID, independently of anemia [17]. In addition, Maeder et al. suggested that the sympathetic activation found in HF patients may contribute to iron depletion, specifically in heart, since Nor stimulation may reduce the intracellular iron levels in cardiac cells by downregulating the transferrin receptor 1 (*Tfr1*) expression [18]. Other studies have shown that iron content in myocardial was reduced by 20–30% in patients with advanced HF [21]. Apart from its role in oxygen transport, iron plays an important role in cellular metabolism, impacting on the regulation of oxidative stress and ATP synthesis, as an enzymatic cofactor in the mitochondria [19, 22]. Radical oxygen species (ROS) directly impacts on the mitochondrial Ca^2+^ homeostasis contributing to an altered contractibility and increasing the cardiac wall stiffness [23]. Hence, iron homeostasis is crucial for cells with high energy demand, such as cardiomyocytes [10, 19].

In spite of all the accumulating data, the interplay of iron in the pathophysiology of HF is not well-defined. We hypothesized that neurohormonal activation could be involved in the generation of ID in cardiac cells, directly impacting on the mitochondria function. Therefore, the aim of this study was to analyze the relationship between neurohormonal stimuli and iron homeostasis, as well as their role regulating the mitochondrial function in cardiac cells.

## Results

We designed a two-step experimental approach. First, the effect of HF over several molecules involved in iron regulation was evaluated in hearts from a well-established isoproterenol-induced HF mice [24]. Second, the mechanisms linking iron regulation, energy metabolism and mitochondrial function, were further explored in depth in heart-derived cell cultures H9c2 exposed to the neurohormonal activation typically found in HF.

### Changes in the gene expression and protein levels of iron regulatory molecules in the heart of isoproterenol-induced HF mice

**Fig. 1:**
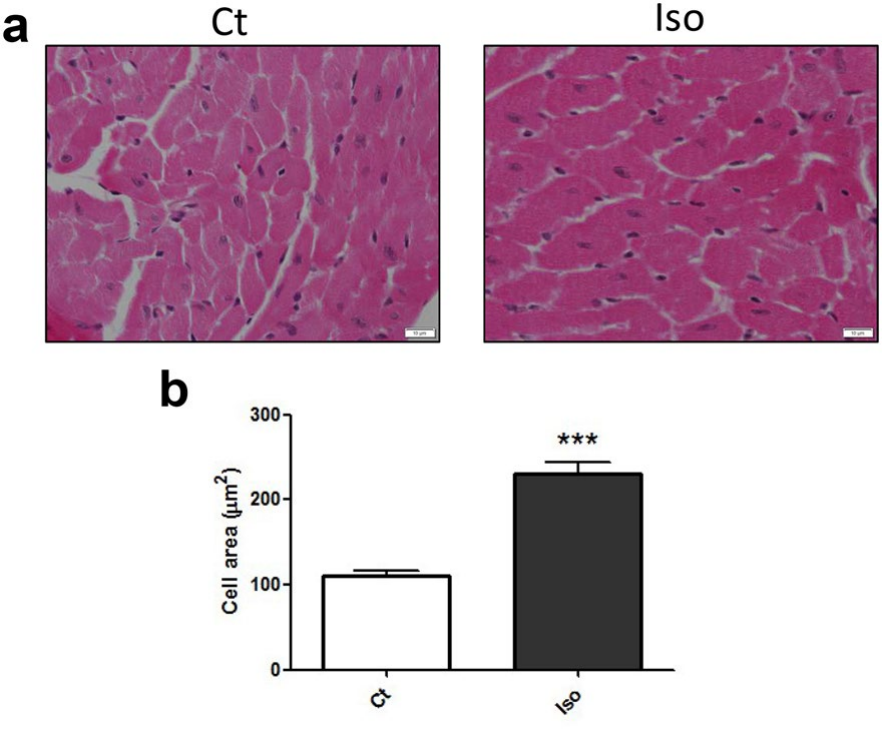
Isoproterenol induces cardiac hypertrophy. **a** Masson’s Trichrome staining of sections from ventricular myocardial of control (Ct) and isoproterenol (Iso) stimulated mice. **b** Quantification of cardiomyocytes area. Data were expressed as mean ± SEM (n = 8 per group) (***p < 0.001 vs control)

First, we analyzed the cardiomyocyte area on histological sections from mice hearts (Fig. 1a). As it is shown in the Fig. 1, isoproterenol induced cellular hypertrophy, compared to control animals (Fig. 1a, b).

**Fig. 2:**
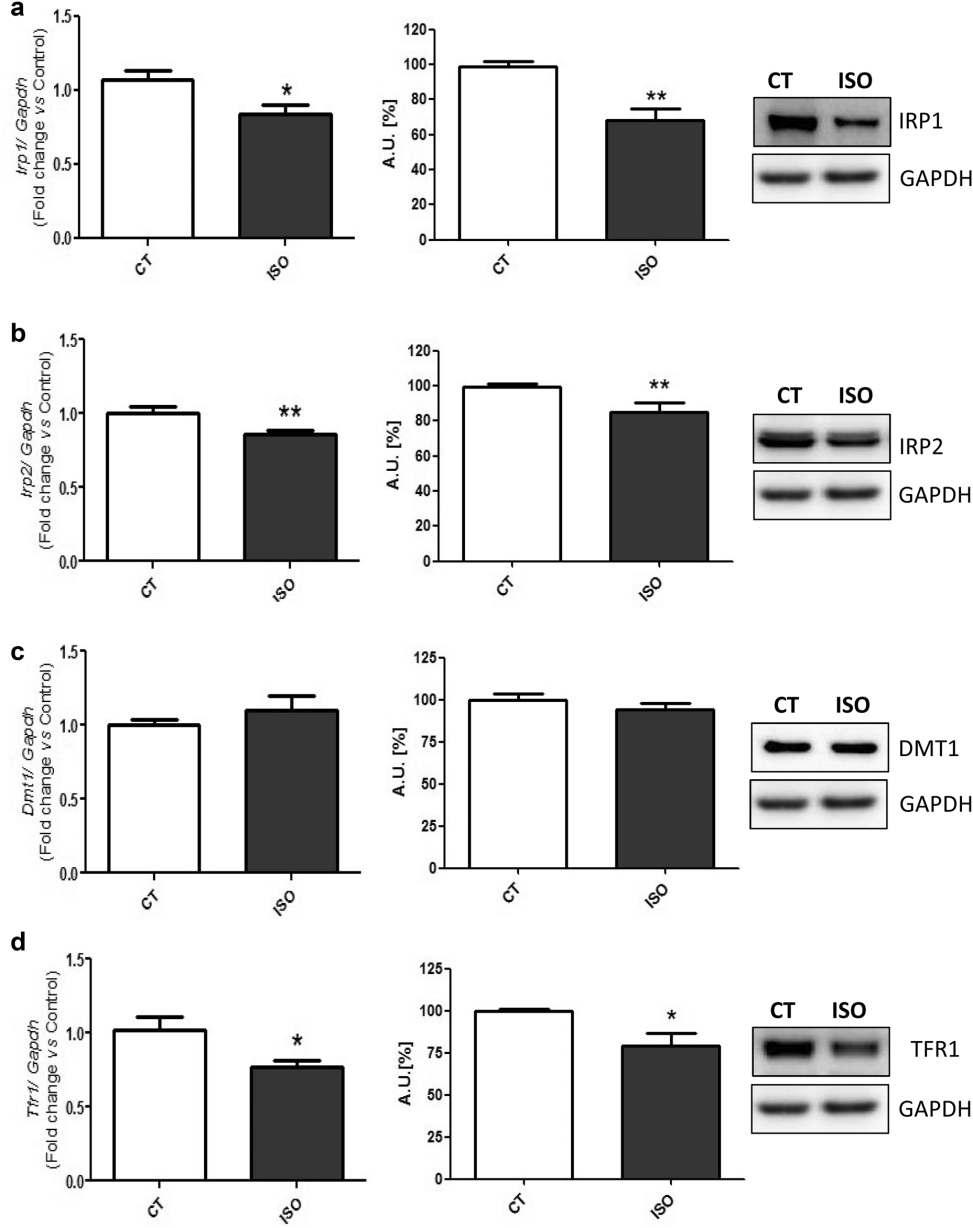
Isoproterenol-induced HF mice heart presents changes in iron regulatory molecules. Analysis of (**a**) Irp1 mRNA and protein levels; **b** Irp2 mRNA and protein levels; **c** Dmt1 mRNA and protein levels; and **d** Tfr1 mRNA and protein levels. Representative Western Blot image of all the analyzed proteins. Data were normalized by the Gapdh mRNA or protein levels and expressed as mean ± SEM (n = 7 per group for mRNA; n = 5 per group for protein); (*p < 0.05 and ** p < 0.01 vs control)

Both, the mRNA and protein levels of the main molecules related to cellular iron uptake, release and storage, were analyzed in HF and control mice. First, it was determined the two iron regulatory proteins (Irp1 and 2), which regulate the translation of several proteins involved in iron metabolism [21]. Irp1 (Fig. 2a) and 2 (Fig. 2b) were down-regulated in mice stimulated with isoproterenol compared to control animals. Next, the levels of the main iron transporters into the cell, Tfr1 and divalent metal transporter 1 (Dmt1), were also determined. Whereas Dmt1 was unaltered (Fig. 2c), Tfr1 was reduced in the hearts of HF mice (Fig. 2d).

**Fig. 3:**
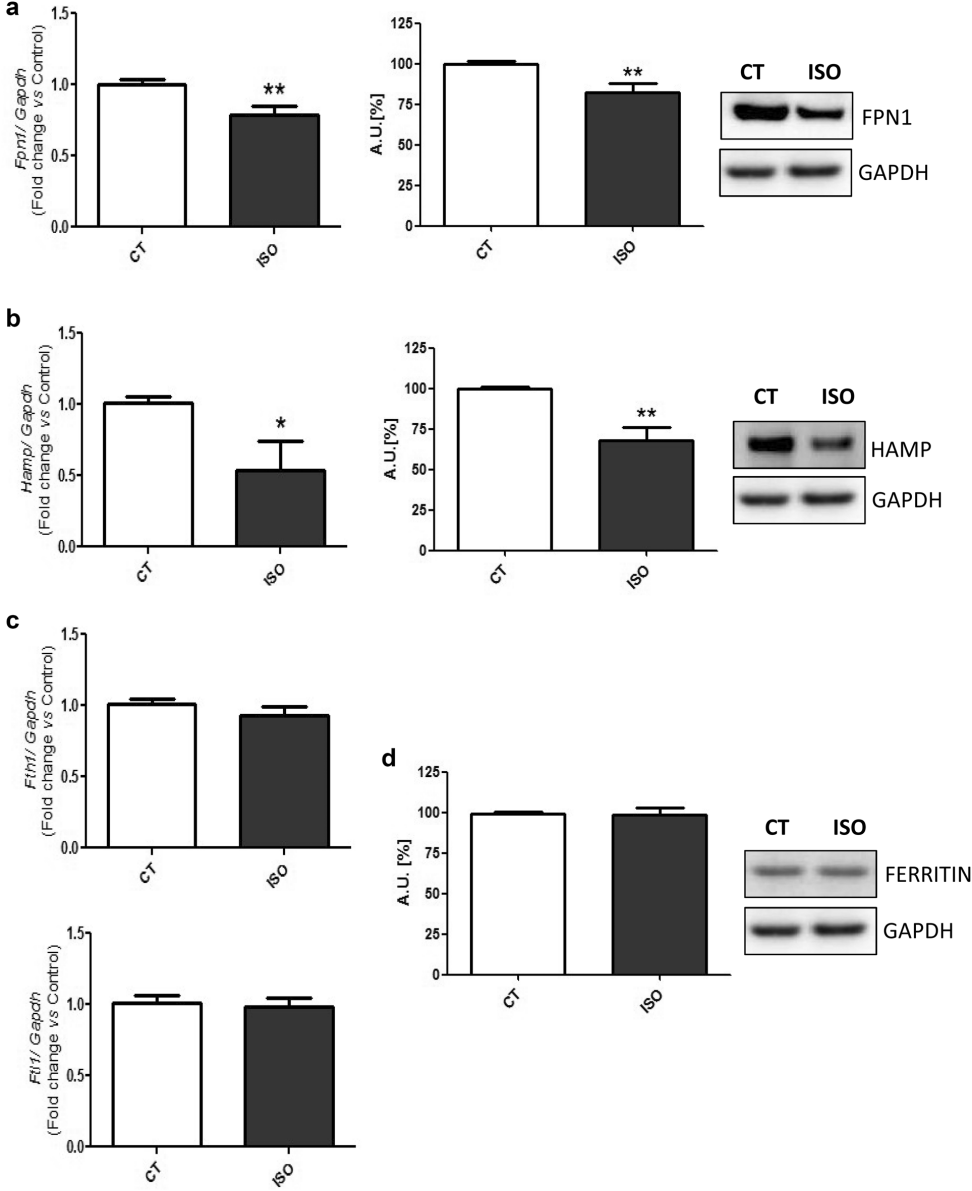
Isoproterenol-induced HF mice heart presents changes in release and storage iron regulatory molecules. Analysis of (**a**) Fpn1 mRNA and protein levels, **b** Hamp mRNA and protein levels; **c** *Fth1* and *Ftl1* mRNA levels; and **d** Ferritin protein levels. Representative Western Blot image of all the analyzed proteins. Data were normalized by the Gapdh mRNA or protein levels and expressed as mean ± SEM (n = 7 per group for mRNA; n = 5 per group for protein); (*p < 0.05 and **p < 0.01 vs control)

In order to look deeper into the role of the molecules related to the cellular iron release, ferroportin 1 (Fpn1) (Fig. 3a), and its inhibitor, hepcidin antimicrobial peptide (Hamp) (Fig. 3b) were analyzed. In the isoproterenol-stimulated mice, the levels of both molecules were down-regulated when compared to control animals. Finally, the cytoplasmic iron storage molecule ferritin, encoded by ferritin heavy chain 1 (*Fth1*) and ferritin light chain 1 (*Ftl1*), was analyzed. No changes were found between the experimental groups in the *Fth1* and *Ftl1* mRNA (Fig. 3c), neither in the Ferritin protein levels (Fig. 3d).

### Regulation of mitochondrial iron metabolism molecules in the heart of HF mice

**Fig. 4:**
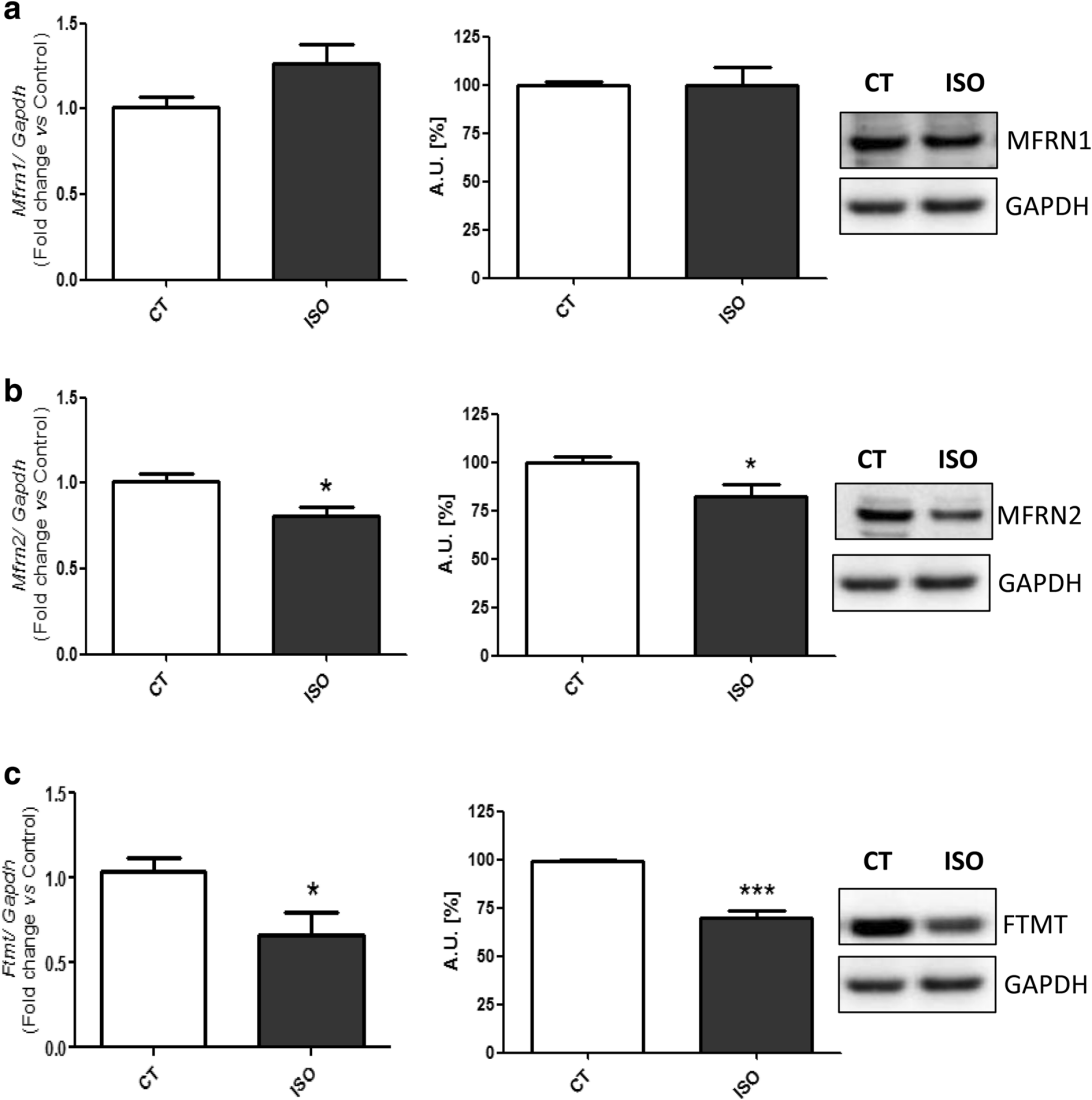
Isoproterenol-induced HF mice myocardial tissue presents changes in mitochondrial iron regulatory molecules. Analysis of (**a**) Mfrn1 mRNA and protein levels, **b** Mfrn2 mRNA and protein levels, **c** Ftmt mRNA levels and protein levels. Representative Western Blot image of all the analyzed proteins. Data were normalized by the Gapdh mRNA protein levels and expressed as mean ± SEM (n = 7 per group for mRNA; n = 5 per group for protein); (*p < 0.05 and *** p < 0.001 vs control)

Due to the role of iron in mitochondrial function, the mitochondrial iron uptake transporters Mitoferrin 1 (Mfrn1) (Fig. 4a) and 2 (Mfrn2) (Fig. 4b), and the mitochondrial iron storage molecule Mitochondrial ferritin (Ftmt) (Fig. 4c) were analyzed. Whereas the Mfnr1 was not modified, the Mfrn2 was down-regulated in the hearts from the HF animals. In addition, Ftmt was also down-regulated in hearts from isoproterenol-stimulated mice.

### Intracellular iron depletion in the heart of isoproterenol-induced HF mice

**Fig. 5:**
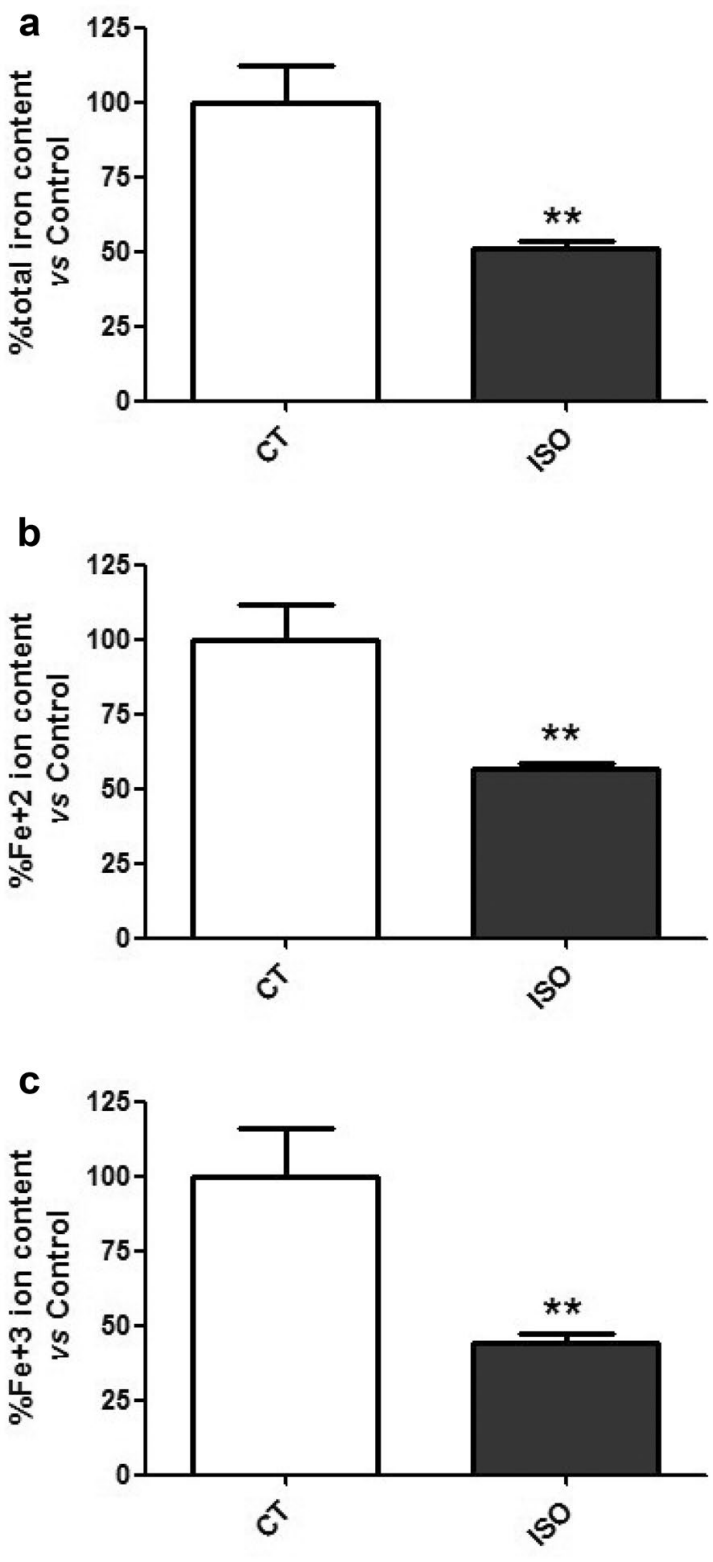
Iron content depletion in the myocardial tissue of isoproterenol-induced HF mice. Analysis of intracellular iron levels in hearts: (**a**) total iron content, **b** Fe^2+^ ion content and **c** Fe^3+^ ion content. Data were normalized by protein levels and expressed as mean ± SEM (n = 5 per group); (**p < 0.01 vs control)

Finally, the total heart iron (Fig. 5a), as well as the iron ions Fe^2+^ (Fig. 5b) and Fe^3+^ (Fig. 5c), were analyzed in mice. As it is shown in Fig. 5, the iron ions were reduced in the hearts from the animals stimulated with isoproterenol compared to control ones.

### Neurohormonal stimulation induced hypertrophy in H9c2 cells

**Fig. 6:**
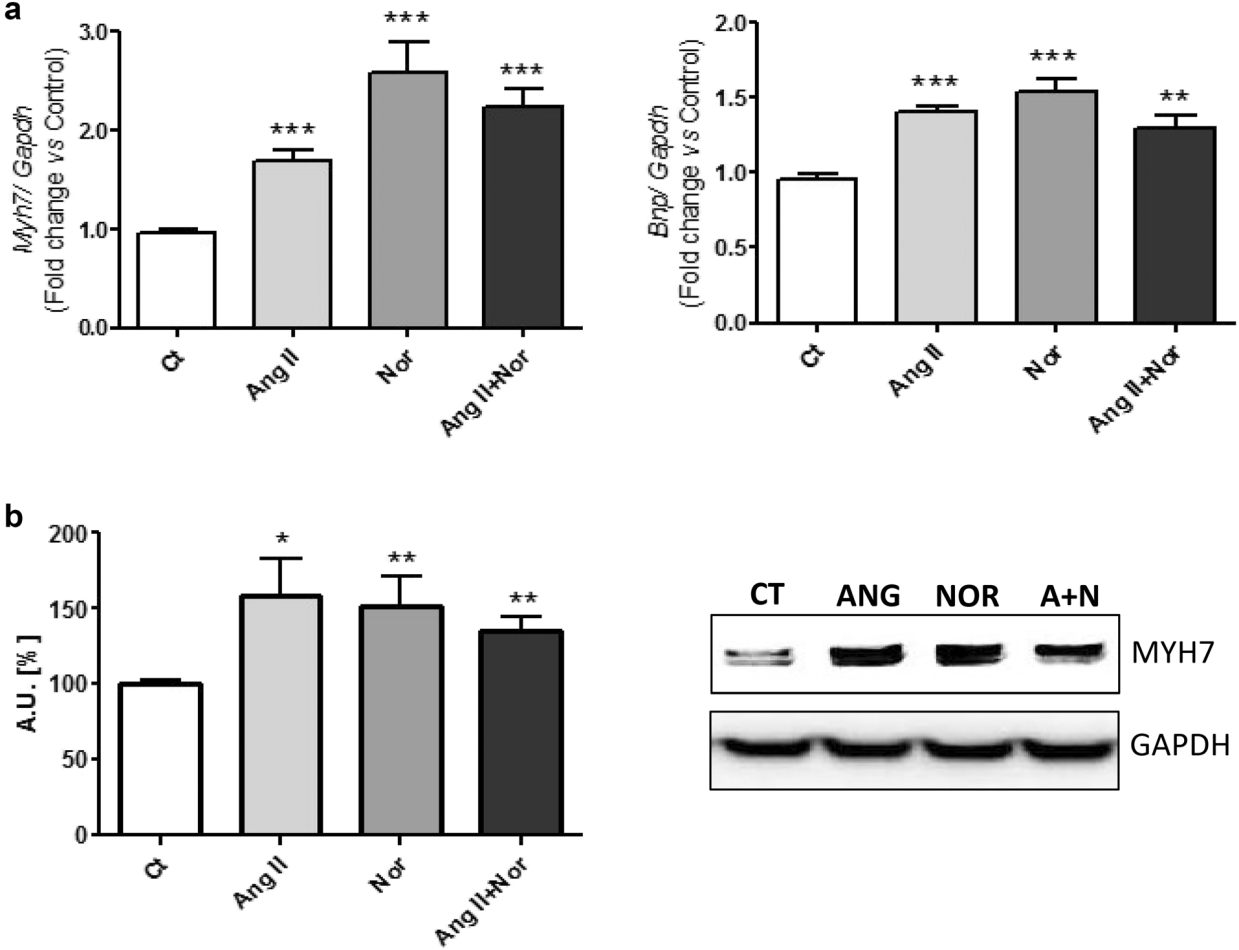
Neurohormonal activation induces hypertrophy in H9c2 cells. Analysis of (**a**) *Myh7* and *Bnp* mRNA levels. **b** Western blot showing MYH7 protein levels. Data were normalized by the Gapdh mRNA (**a**) or protein (**b**) levels and expressed as mean ± SEM (n = 7 per group); (*p < 0.05, **p < 0.01 and *** p < 0.001 vs control)

To further explore the role of neurohormonal activation in iron metabolism at cellular level, an in vitro cardiac cell model was studied. Since myocardial hypertrophy is a hallmark of HF [3], some of the classical markers of cardiac hypertrophy (Bnp and Myh7) [25] were assessed in the H9c2 cells stimulated with the neurohormonal stimuli (Ang II and/or Nor). As it is shown in Fig. 6, both Ang II and Nor induced an increase in the mRNA levels of *Bnp* and *Myh7* (Fig. 6a). Similar results were found when the MYH7 protein levels were analyzed (Fig. 6b).

### Neurohormonal activation induced changes in the intracellular iron metabolism molecules in H9c2 cells

**Fig. 7:**
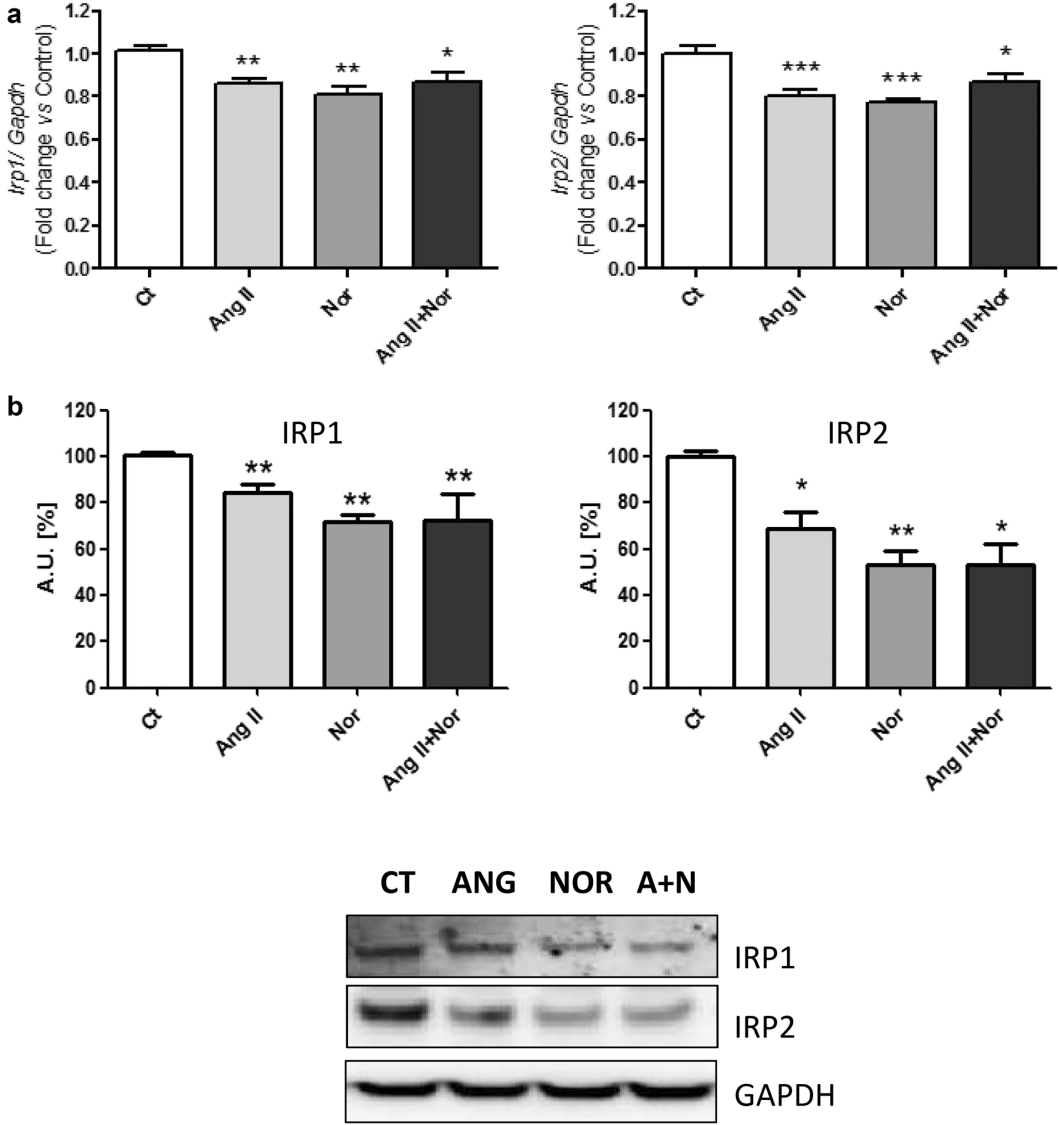
Neurohormonal activation modifies iron metabolism related molecules in H9c2 cells. Analysis of (**a**) *Irp1* (left) and *Irp2* (right) mRNA levels and (**b**) IRP1 (left) and IRP2 (right) protein levels. Representative Western Blot image of all the analyzed proteins. Data were normalized by the *Gapdh* mRNA (**a**) or protein (**b**) levels and expressed as mean ± SEM (n = 7 per group); (*p < 0.05, **p < 0.01 and *** p < 0.001 vs control)

**Fig. 8:**
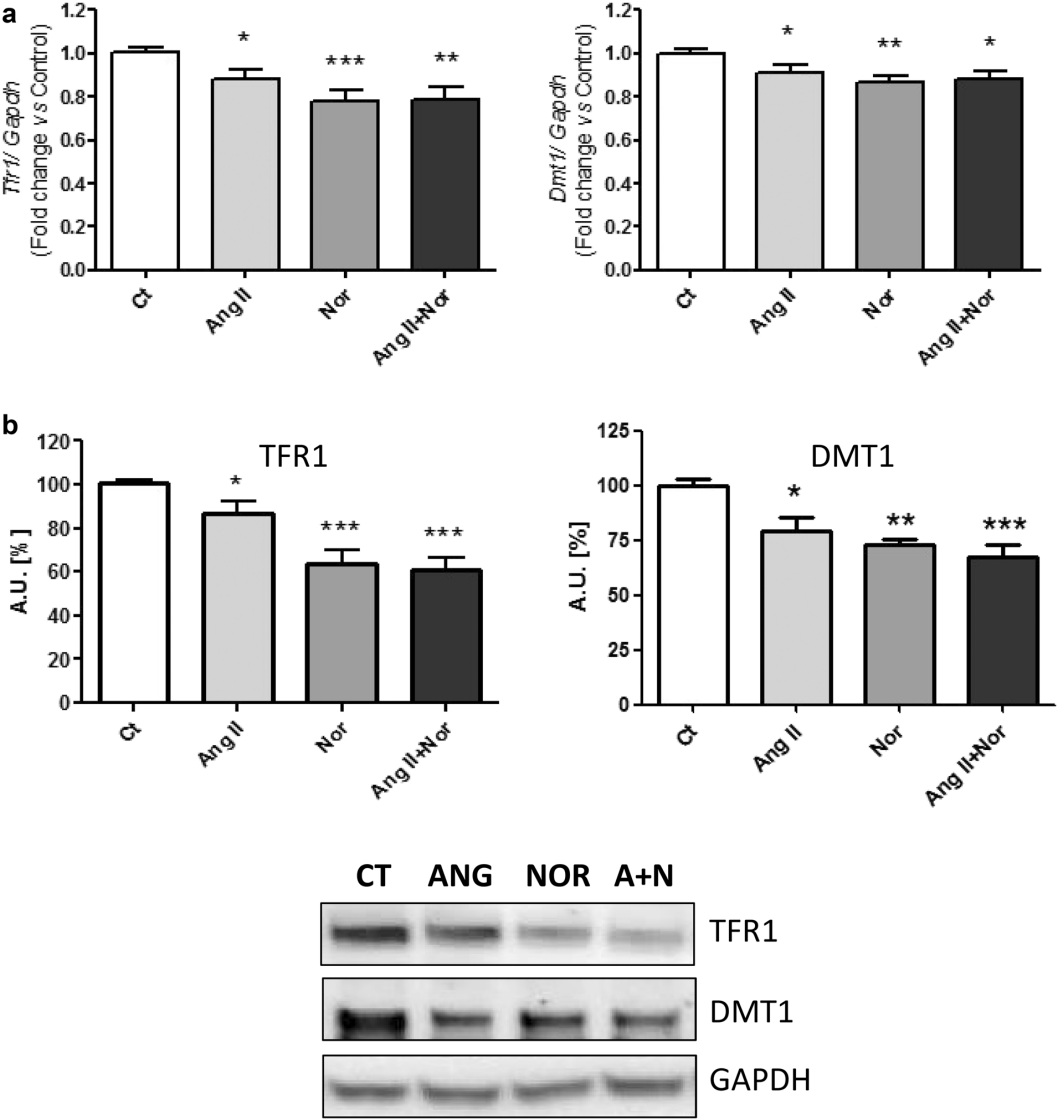
Neurohormonal activation modifies iron uptake molecules in H9c2 cells. Analysis of (**a**) *Tfr1* (left) and *Dmt1* (right) mRNA levels; **b** TFR1 (left) and DMT1 (right) protein levels. Representative Western Blot image of all the analyzed proteins. Data were normalized by the Gapdh mRNA (**a**) or protein (**b**) levels and expressed as mean ± SEM (n = 7 per group); (*p < 0.05, **p < 0.01 and ***p < 0.001 vs control)

The same molecules studied in mice were subsequently assessed in the in vitro neurohormonal activation model. Irp1 and 2 were significantly reduced when cells were exposed to all the experimental conditions (Fig. 7a, b). In addition, Tfr1 and Dmt1 were down-regulated in the setting of any of the neurohormonal challenges (Fig. 8a, b).

**Fig. 9:**
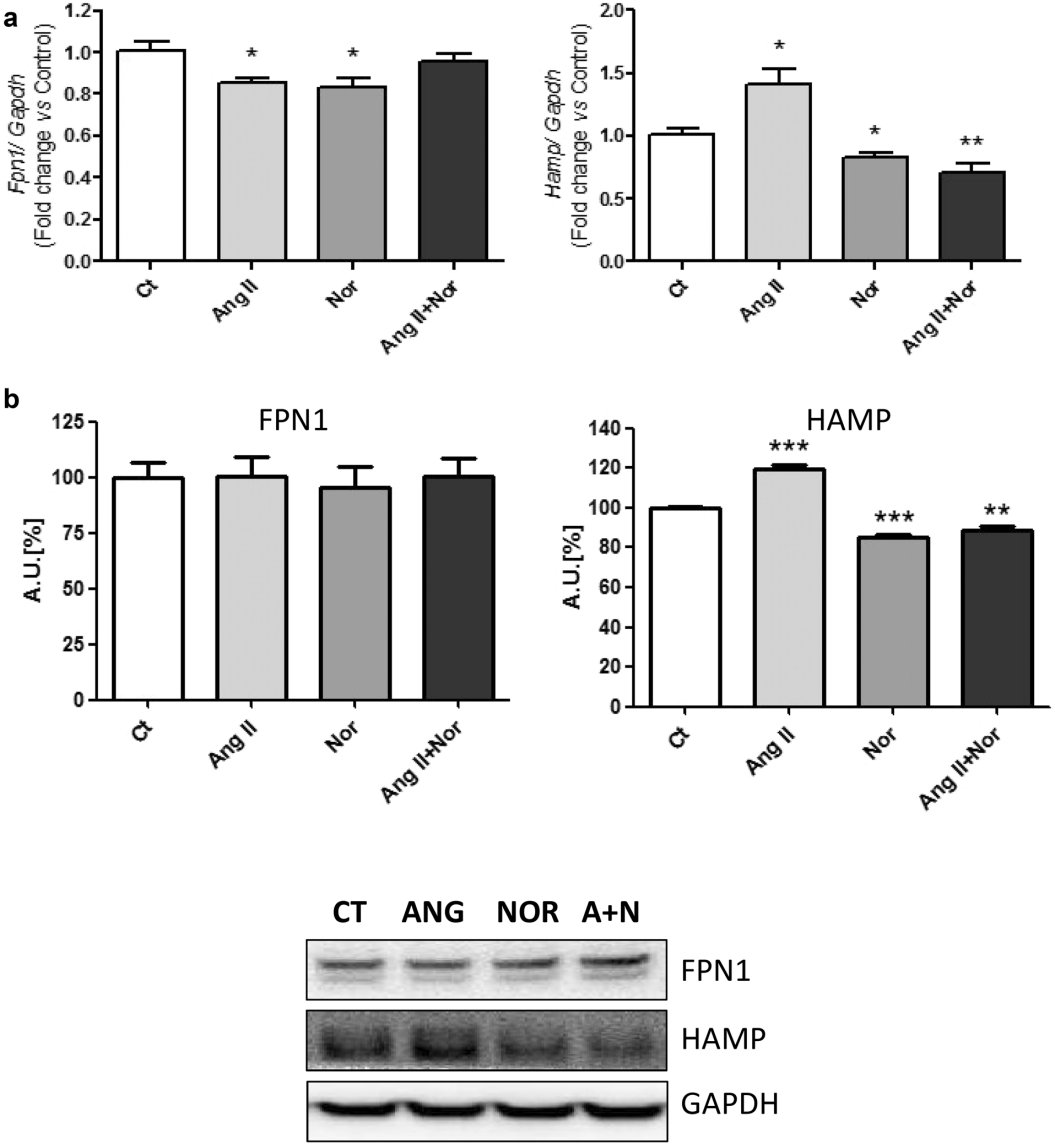
Neurohormonal activation modifies iron released molecules in H9c2 cells. Analysis of (**a**) *Fpn1* (left) and *Hamp* (right) mRNA levels; (b) FPN1 (left) and HAMP (right) protein levels. Representative Western Blot image of all the analyzed proteins. Data were normalized by the Gapdh mRNA (**a**) or protein (**b**) levels and expressed as mean ± SEM (n = 7 per group); (*p < 0.05, **p < 0.01 and ***p < 0.001 vs control)

With respect to cellular iron release, whereas *Fpn1* mRNA levels were reduced under Ang II or Nor treatment alone, there were no changes in its expression when cells were simultaneously treated with both stimuli (Fig. 9a, left). Fpn1 protein levels did not change in any experimental condition (Fig. 9b, left). On the other hand, while Hamp was induced by Ang II treatment, its levels were reduced under the influence of Nor and simultaneous treatment with Nor and Ang II (Fig. 9a, right and Fig. 9b right).

**Fig. 10:**
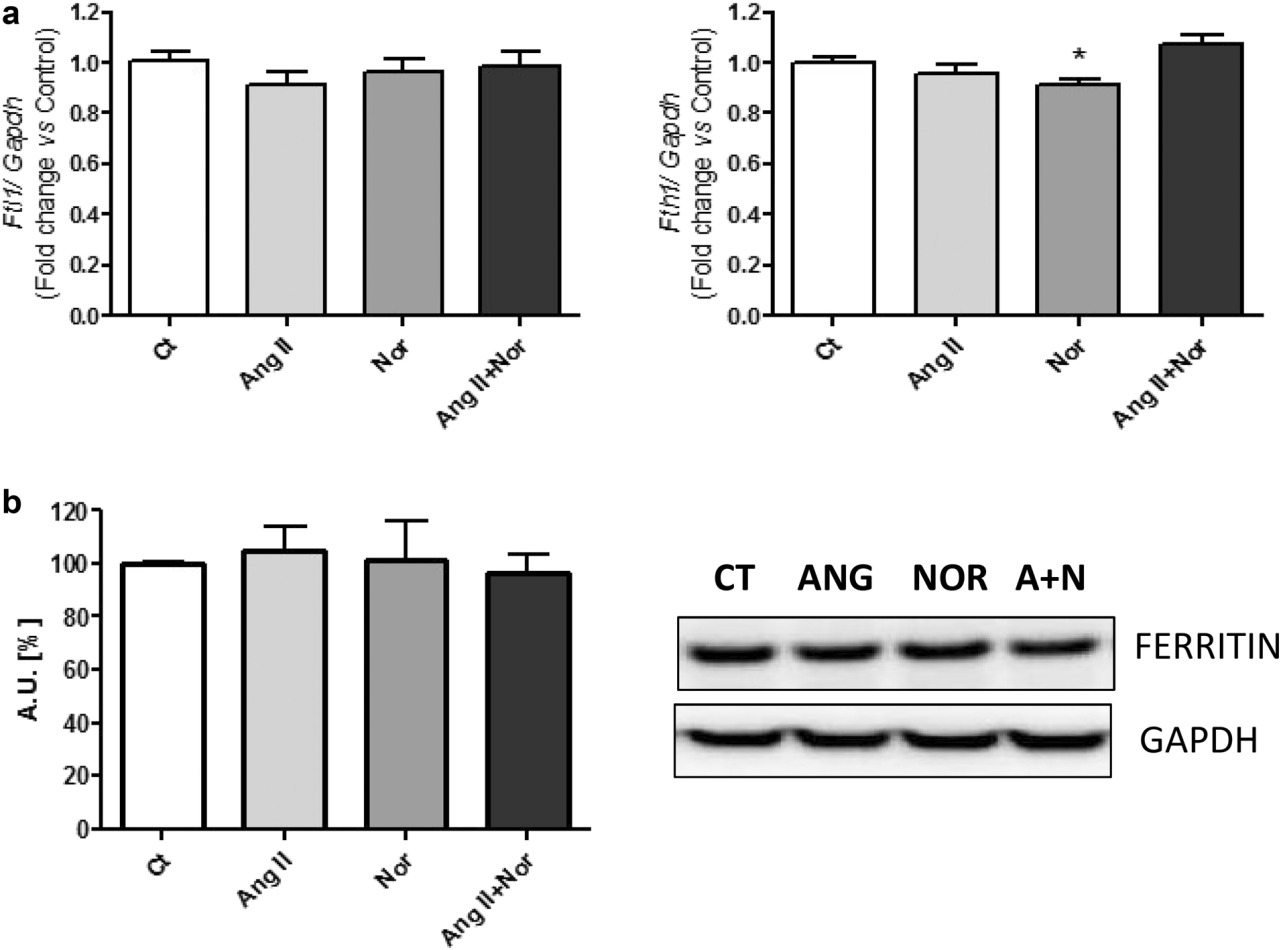
Neurohormonal activation does not modify ferritin in H9c2 cells. Analysis of (**a**) *Ftl1* (left) and *Fth1* (right) mRNA levels; (**b**) Ferritin protein levels. Representative Western Blot image of all the analyzed proteins. Data were normalized by the Gapdh mRNA (**a**) or protein (**b**) levels and expressed as mean ± SEM (n = 7 per group); (*p < 0.05 vs control)

Finally, although the stimulation with Nor produced a slight reduction in the *Fth1* expression, there were no significant changes when exposing cardiac cells to Ang II or both stimuli at the same time (Fig. 10a, right). Also, no significant changes in *Ftl1* expression were found in any experimental condition (Fig. 10a, left). Accordingly, no significant changes were found in the cytoplasmic Ferritin protein levels (Fig. 10b).

### Neurohormonal activation depleted intracellular iron in H9c2 cells

**Fig. 11:**
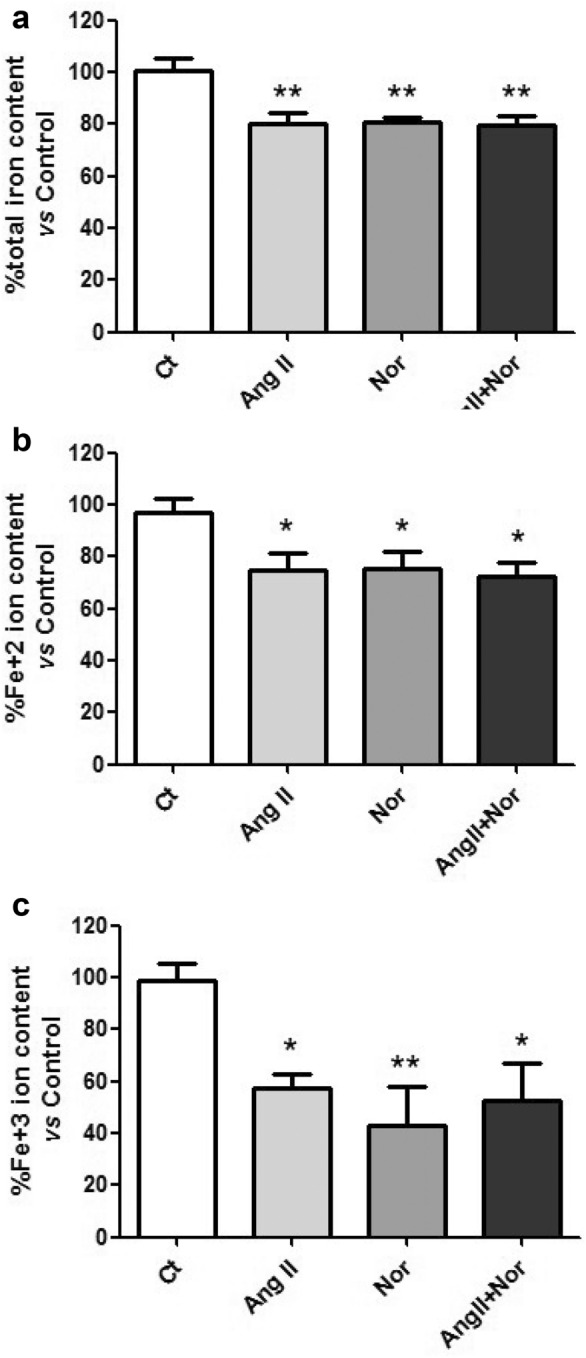
Neurohormonal activation depletes intracellular iron in H9c2 cells. Analysis of intracellular iron levels: (**a**) total iron content, **b** Fe^2+^ ion content and (c) Fe^3+^ ion content. Data were normalized by protein levels and expressed as mean ± SEM (n = 7 per group); (*p < 0.05 and **p < 0.01 vs Control)

Beyond the effects of neurohormonal activation on the molecules involved in iron metabolism, its effect on the intracellular iron levels was assessed in H9c2 cells. As it is shown in Fig. 11, the total iron (Fig. 11a), as well as the iron ions Fe^2+^ (Fig. 11b) and Fe^3+^ (Fig. 11c), were reduced in the cells challenged with Nor, Ang II, or their combination, when compared to control cells.

### Neurohormonal activation promoted down-regulation of Ftmt levels in H9c2 cells

**Fig. 12:**
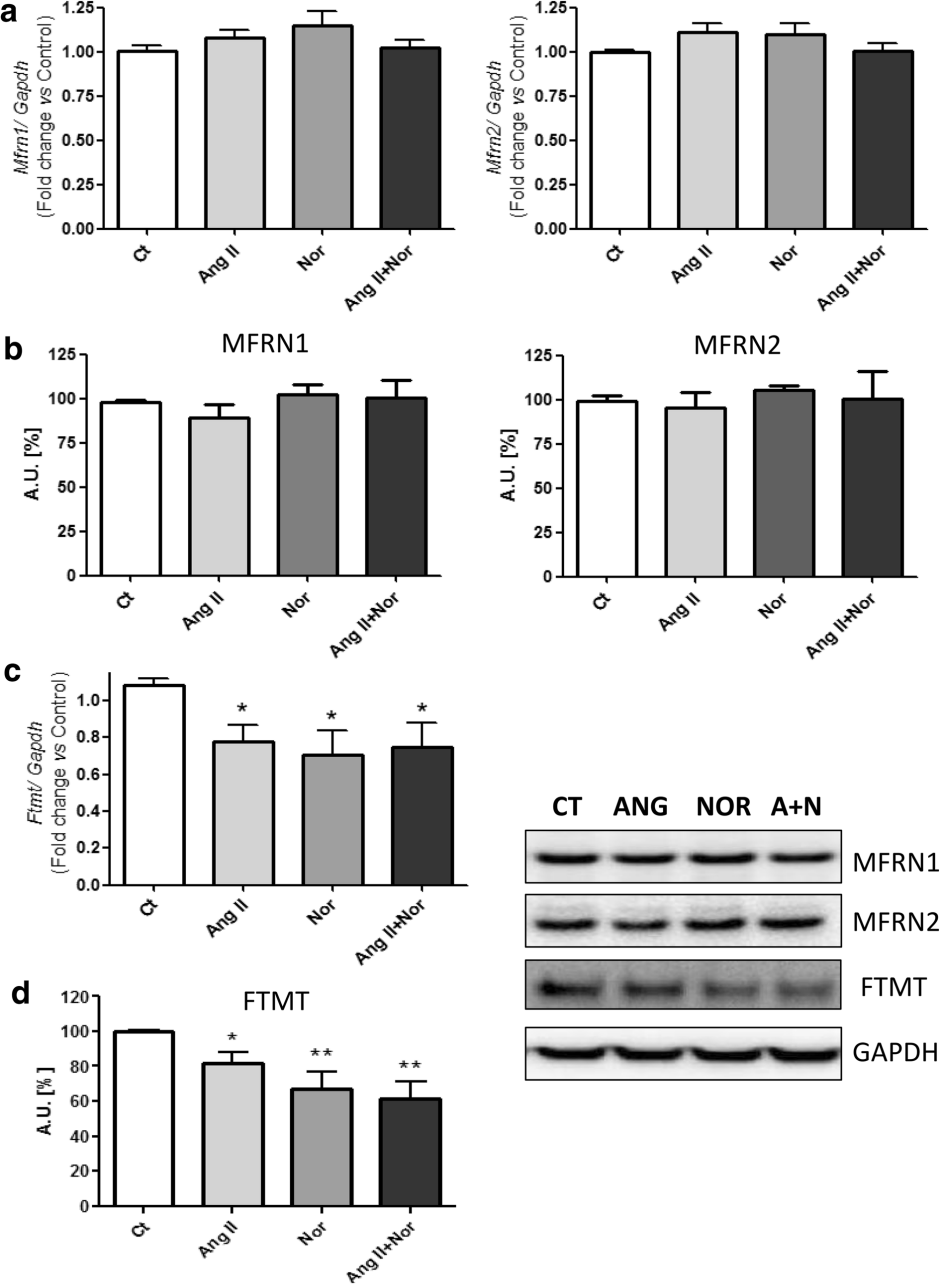
Neurohormonal activation modifies mitochondrial iron storage related molecules in H9c2 cells. Analysis of (**a**) *Mfrn1* and *Mfrn2* mRNA levels, (**b**) MFRN1 and MFRN2 protein levels, (**c**) *Ftmt* mRNA levels and (d) FTMT protein levels. Representative Western Blot image of all the analyzed proteins. Data were normalized by the Gapdh mRNA (**a**, **c**) or protein (**b**, **d**) levels and expressed as mean ± SEM (n = 7 per group); (*p < 0.05 and **p < 0.01 vs Control)

The exposure to Nor and/or Ang II did not induce changes in the levels of the mitochondrial iron transporters Mfrn1 and Mfrn2 (Fig. 12a, b). Interestingly, the mRNA and protein levels of the Ftmt were reduced in all the experimental conditions (Fig. 12c, d).

### Neurohormonal activation impaired mitochondrial function in H9c2 cells

**Fig. 13:**
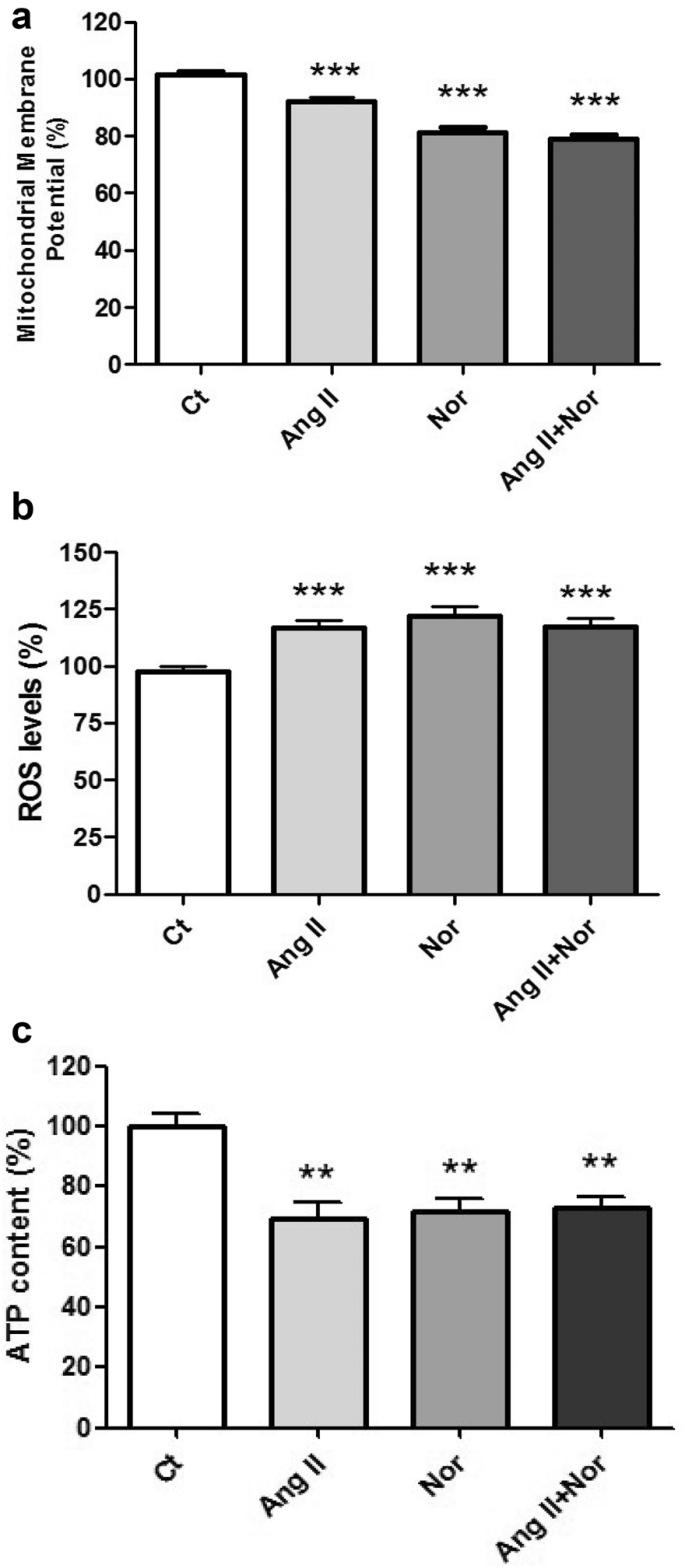
Neurohormonal activation impaires mitochondrial function. Quantification of different parameters: (**a**) Mitochondrial membrane potential, **b** intracellular ROS production, and (**c**) ATP levels. Data were normalized by the cellular protein content and expressed as mean ± SEM (n = 7 per group); (**p < 0.01 and *** p < 0.001 vs control)

Several parameters were assessed to characterize mitochondrial function in the neurohormonal activation in vitro model. In the stimulated cells, the mitochondrial membrane potential was reduced (Fig. 13a). Additionally, there was an increase in the ROS production (Fig. 13b), along with a decrease in the ATP levels (Fig. 13c), regardless of the type of stimuli.

## Discussion

ID has been associated with adverse outcomes in HF [26, 27]. Despite the clinical association between HF and ID, it is currently unknown whether intracellular ID in HF-cardiac cells is a consequence of the disease or, otherwise, directly takes part in the cellular alterations leading the pathology. In the present study we demonstrated that neurohormonal activation related to HF promoted significant abnormalities in the iron metabolism regulatory systems, reduced intracellular iron levels and impaired mitochondrial function in cardiac cells.

Although the unequivocal benefits of intravenous iron may be related to a peripheral effect [14, 28], recent studies support a cardiac-centered hypothesis. First, ferric carboxymaltose administration was associated with cardiac iron repletion in cardiac magnetic resonance T2* and T1 mapping sequences [29]. Second, Doppler and strain rate echocardiography parameters were significantly improved after intravenous iron administration in patients with stable systolic HF and ID without anemia [30]. Third, recent data in patients with HF have correlated ID and reduced peak exercise capacity with impaired myocardial contractile reserve [31].

We set up a study with both in vivo and in vitro approaches. First, we used a well-established HF mice model induced by isoproterenol (β-adrenoceptor agonist) osmotic pumps [24, 32]. Interestingly, these animals showed an increase of Ang II in blood and heart [33], thereby being a good model to study the effect of neurohormonal activation. Moreover, the results found in the in vivo model were validated in H9c2 cardiac cells challenged with AngII, Nor or both, in order to mimic the neurohormonal activation with more than just on stimuli [3, 6].

To validate the neurohormonal activation cell model, we analyzed the expression of cardiac hypertrophy molecules (Bnp and Myh7) as hallmarks of HF [3, 34]. Our neurohormonal activation in vitro model induced both mRNA and protein levels of cardiac hypertrophy markers in all the experimental conditions. Nevertheless, no synergistic effect was observed in cells stimulated with both stimuli simultaneously, possibly because the stimulation with each of them separately already achieved the maximum effect. Similarly, isoproterenol also induced cardiomyocyte hypertrophy in mice.

Our data showed that the neurohormonal activation was related to a reduction of the intracellular iron levels (both Fe^2+^ and Fe^3+^), thereby suggesting that neurohormonal stimuli may contribute to cardiac alteration through intracellular ID. Interestingly, previous studies reported iron depletion in myocardial tissue of HF patients [18, 22] and highlighted the relevance of neurohormonal activation leading to iron depletion.

At a cardiac cell level, ID may be due to several mechanisms involving iron uptake, release and storage. Interestingly, Irps are able to regulate the translation of different molecules related to these processes depending on iron levels [35]. As a matter of fact, Haddad et al*.* showed that Irps are crucial to maintain the iron supply in cardiomyocytes and to prevent HF in mice [21]. Furthermore, the same study showed a decrease in the Irp activity from HF patients, related to a depletion of iron in the heart [21]. Accordingly, our study evidenced a down-regulation of the Irps in both of our experimental models. Next, we assessed the levels of Tfr1, which is involved in the tranferrin-bound iron intake [36]. Xu et al. showed that the lack of Tfr1 in knock-out mice produced a lethal cardiomyopathy by diminishing the intracellular iron content and impairing mitochondrial function [37]. Besides, the levels of Tfr1 were found reduced in the myocardial tissue of HF patients [18, 21]. Interestingly, Maeder et al. demonstrated that cardiomyocytes treated with aldosterone or Nor showed a decrease in the Tfr1 levels [18]. In line with these studies, we found that Tfr1 was decreased in both hearts of HF mice and in the cardiac cells, in the setting of any of the neurohormonal challenges. Finally, although the Dmt1, related to the non-transferrin bound iron uptake [38], was slightly down-regulated in the neurohormonal-stimulated cells, we found no modifications in hearts from isoproterenol-induced HF mice. As Kobak et al*.* recently reviewed, Dmt1 uptake could act as a compensatory mechanism of the Tfr1-mediated iron import [39].

Neurohormonal stimuli also regulate the molecules involved in the cellular iron release, Fpn1 and Hamp. In the heart, Hamp participates in the local iron homeostasis regulation by inhibiting Fpn1 function [40]. Fpn1 exports intracellular iron outside the cell [41]. Deregulations that affect the homeostasis of the Fpn1/Hepcidin axis are known to produce iron overload or anemia, depending on the direction of the functional changes [42]. In the studied models we found different behaviors when analyzing these components. Whereas *Fpn1* mRNA levels were reduced under AngII and Nor stimulation, the combination of both stimuli not induces changes in the expression of this gene in cardiac cells. In contrast, in the isoproterenol treated mice, we observed a decreased of Fpn1 compared to the control mice. On the other hand, Hamp was downregulated in isoproterenol-stimulated mice, as well as in cells challenged with Nor and both stimuli together. Therefore, although the Fpn1 levels decrease in the HF mice model, probably as an attempt to stop the iron release, the reduced Hamp levels could favor the release of iron by Fpn1. Nevertheless, Hamp was upregulated in the cells challenged only with Ang II. Interestingly, the expression of Hamp is up-regulated in iron-deprived environments to secure the intracellular iron content by exerting a negative feed-back on Fpn1 in the cardiomyocytes [40]. Our data suggest that while Ang II-stimulated cells would try to retain the intracellular iron by increasing the levels of Hamp, the exposure to a more intense neurohormonal insult would counteract this compensatory mechanism, and could favored the iron release. To note, the *Hamp* knock-out mice induce myocardial ID and dilated cardiomyopathy due to an increase of iron release [42].

On the other hand, our study did not evidence changes in the cytoplasmic iron storage molecule ferritin by the neurohormonal activation stimuli (in any experimental condition). It is worth to note that ferritin may be further regulated by mechanisms others than Irps, such as oxidative stress [43].

Altogether, our results suggest that neurohormonal activation may contribute to intracellular iron depletion by both increasing intracellular iron release and reducing extracellular iron uptake in cardiac cells. It means that the neurohormonal activation observed in HF patients could participate in disease progression through intracellular iron deprivation. However, further studies should be conducted to show the exact mechanisms underlying the neurohormonal regulation of these components in the failing heart.

The role of iron in cardiac function is closely related to the mitochondria because of its role as an enzymatic cofactor, and the role of mitochondrial dysfunction in HF is being increasingly recognized [44, 45]. Due to the importance of mitochondria in cardiomyocytes, we assessed the levels of the mitochondrial iron uptake transporter Mfrn and the iron storage molecule Ftmt. Mfrn1 and Mfrn2 were not altered in the neurohormonal-stimulated cells, although in hearts from isoproterenol-induced mice the Mfrn2 was down-regulated. Paradkar et al*.* showed that the reduction in *Mfrn1* and *Mfrn2* by RNA interference resulted in a decreased mitochondrial iron accumulation, heme synthesis and iron-sulfur cluster synthesis [46]. Interestingly, Mfrn2 is more ubiquitously expressed than Mfrn1. Besides, we observed a down-regulation of the Ftmt levels in all the experimental conditions. Our data are in line with those reported in hearts from Ftmt^−/−^ mice, since these animals showed mitochondrial damage and fibril disorganization [40]. Moreover, given the antioxidant role of Ftmt [47], neurohormonal activation may directly contribute to make cardiac cells more sensitive to oxidative stress. This hypothesis is further supported by other studies showing the decrease of antioxidant enzymes in hearts from HF patients [22].

Our data revealed that neurohormonal activation increased ROS production and reduced mitochondrial membrane potential in cardiac cells, showing impairment in the mitochondria. Mitochondria are crucial to maintain the high-energy demand of the heart [10, 22]. Actually, mitochondrial function has been found impaired in myocardial tissue of advanced HF patients [10]. The role of myocardial and cardiomyocyte iron depletion leading to mitochondrial malfunction has been highlighted as one of the drivers impairing contractile function [19]. Accordingly, mitochondrial dysfunction found in the cells challenged with the neurohormonal stimuli was related to a decrease in the ATP levels. Altogether, our results are in line with the data reviewed by Brown et al. suggesting that mitochondrial impairment could be linked to cardiomyocyte injury and HF progression [45].

**Fig. 14:**
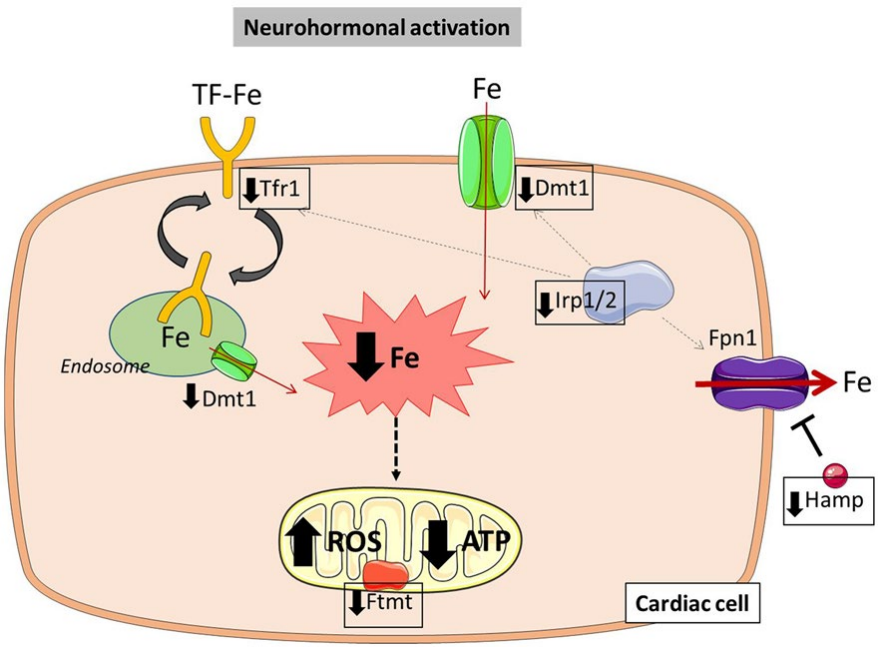
Changes in iron metabolism due to neurohormonal activation in cardiac cells. This illustration describes the key iron molecules modified under neurohormonal activation in cardiac cells. It is also defined the iron deficiency and the mitochondrial dysfunction present under the neurohormonal stimuli. *ATP* adenosine triphosphate, *Dmt1* divalent metal transporter 1, *Fe* iron, *Fpn1* ferroportin 1, *Ftmt* mitochondrial ferritin, *Hamp* hepcidin, *Irp1/2* iron regulatory proteins ½, *ROS* reactive oxygen species, *Tf-Fe* transferrin-bound iron, *Tfr1* transferrin receptor 1

Finally, Fig. 14 summarized the key results obtained in this work, showing the effect of neurohormonal activation over the main iron metabolism molecules, the intracellular iron deficiency and the mitochondrial dysfunction at cardiac cell level (Fig. 14).

## Conclusions

In conclusion, our data provide evidences that the neurohormonal activation observed in the setting of HF promotes a reduction in intracellular iron levels within cardiac cells and hampers mitochondrial function. The molecular mechanisms mediating this relationship may involve a transcriptional regulation of several iron metabolism genes in the cell. The current results suggest that ID could be a key element in the pathophysiological sequence that leads to the progression of HF. Nevertheless, further research is necessary to fully characterize the role of iron in the pathophysiology of HF.

## Materials and methods

### Animal model

Sixteen 10-week-old male C57BL/6 mice received a continuous infusion of isoproterenol (ISO group; n = 8) or vehicle (saline) (Control group; n = 8) at a rate of 30 mg/kg/day for 28 days using a subcutaneously implanted osmotic mini-pump (Alzet, model 1004), to generate a validated model of experimental HF in mice [24]. The animals were anesthetized by isoflurane inhalation during the implantation pump. Buprenorphine (0.3 mg/kg, i.p.) was administered 10 min before surgery and after 24 h. Mice were euthanized by an i.p. injection of pentobarbital sodium (100 mg/kg). The experimental protocol was approved by the local Institutional Ethics Committee of the Institut Municipal d’Investigacions Mèdiques-Universitat Pompeu Fabra (CEEA-PRBB 16-1814I) and all animal procedures performed according to the guidelines from Directive 2010/63/EU of the European Parliament on the protection of animals used for scientific purposes.

Heart samples were fixed in buffered 4% paraformaldehyde, embedded in paraffin and cut into 4 µm-thick slices. Heart sections were deparaffinized and rehydrated with xylene, etanol (100%, 90%, 70%) and water, and stained with Masson’s Trichrome Stain Kit (Polyscience) to assess cardiomyocyte hypertrophy. Representative ventricular photomicrographs (5–10 per animal) were acquired at 400 × magnification with a light microscope (BX61, Olympus) and a mounted digital camera (DP72, Olympus). LV cardiomyocyte area was measured in at least 30 random cardiomyocytes by outlining round to cuboidal-shaped nucleated cardiomyocites using the ImageJ software.

### Cell culture model

Embryonic rat heart-derived cells (H9c2 cells) were maintained in a high-glucose Dulbecco's modified Eagle’s medium (4.5 g/l glucose) (DMEM; supplemented with 10% fetal bovine serum, 100 U/ml penicillin and 100 μg/ml streptomycin) at 37 °C and 5% CO_2_. Cells were seeded in multi-well plates and serum deprived in DMEM supplemented with 1% fetal bovine serum, 100 U/ml penicillin and 100 μg/ml streptomycin for 16 h, and then stimulated with 1 µM of Ang II (Sigma Aldrich, Spain) or/and 2 µM of Nor (Sigma Aldrich, Spain) for 48 (n = 7 in each experimental group).

### RNA preparation and quantitative real time reverse transcription-polymerase chain reaction (RT-PCR) analysis

Briefly, levels of mRNA were assessed by RT-PCR. Total RNA was isolated from H9c2 cells and mice heart using Nucleospin RNA II kit (Macherey–Nagel, Spain). RNA was quantified by a NanoDrop 1000 Spectrophotometer (Thermo Scientific, Spain). TaqMan gene expression assays-on-demand (Thermo Scientific, Spain) were used for rat myosin heavy chain 7 (Myh7) (Rn00691731_m1), rat natriuretic peptide B (*Bnp*) (Rn00580641_m1), rat iron regulatory protein 1 (*Irp1*) (Rn00569045_m1) and mouse *Irp1* (Mm01223514_m1), rat iron regulatory protein 2 (*Irp2*) (Rn00575852_m1) and mouse *Irp2* (Mm01179595_m1), rat ferroportin (*Fpn1*) (Rn00591187_m1) and mouse *Fpn1* (Mm01254822_m1), rat hepcidin antimicrobial peptide (*Hamp*) (Rn00584987_m1) and mouse *Hamp* (Mm00519025_m1), rat transferrin receptor 1(*Tfr1*) (Rn01474701_m1) and mouse *Tfr1* (Mm01344485_m1), rat divalent metal transporter 1 (*Dmt1*) (Rn01533109_m1) and mouse *Dmt1* (Mm01308330_s1), rat ferritin heavy chain 1 (*Fth1*) (Rn00820640_g1) and mouse *Fth1* (Mm00850707_g1), rat ferritin light chain 1 *(Ftl1)* (Rn04341729_g1) and mouse *Ftl1* (Mm03030144_g1)*,* rat mitochondrial ferritin *(Ftmt)* (Rn01492073_s1) and mouse *Ftmt* (Mm01268428_s1), rat mitoferrin 1 (*Mfrn1*) (Rn01753423_m1) and mouse *Mfrn1* (Mm00471133_m1), rat mitoferrin 2 (*Mfrn2*) (Rn01411393_m1) and mouse *Mfrn2* (Mm01199497_m1). Glyceraldehyde-3-phosphate dehydrogenase (*Gapdh*) was used as the endogenous control, rat (Rn01775763_g1) and mouse (Mm99999915_g1). The results were normalized to *Gadph*, and relative quantification was performed using the comparative Ct (2-DDCt) method. mRNA levels were expressed as fold induction over control.

### Immunoblotting

Whole protein extracts were collected from H9c2 cells in RIPA buffer (0.1% SDS, 150 mM NaCl, 1% Nonidet P40, 50 mM Tris–HCl, 0.5% deoxycholate) containing phosphatase and protease inhibitors (Roche Diagnostics; Basel, Switzerland) and western blot analyses were performed using antibodies against MYH7, TFR1, FT, HAMP, FTMT and GAPDH from Abcam (USA), and FPN1, IRP1, IRP2, MFRN1, MFRN2 and DMT1 from Thermo Scientific (Spain). Detection was performed using the appropriate horseradish peroxidase (HRP)-conjugated secondary antibody (Dako; Glostrup, Denmark). The bands were visualized using ClarityTM Western ECL Substrate (BioRad, Spain) with the Quantity One software (BioRad, Spain). Differences in the protein levels were expressed as arbitrary units (A.U.) percentage induction over control.

### Intracellular iron determination

The intracellular iron ions content was determined in hearts and H9c2 cells using the colorimetric Iron Assay kit (Abcam, Spain), following the manufacturer’s instructions. All assays were performed in duplicate, and measured on a Tecan Infinite F200 microplate reader. Levels of total iron, as well as the ions Fe^2+^ and Fe^3+^, were expressed relative to untreated controls.

### Mitochondrial membrane potential

The mitochondrial membrane potential was determined using the reagent TMRE-Mitochondrial Membrane Potential Assay Kit (Abcam; Spain). All assays were performed in duplicate, and measured on a Tecan Infinite F200 microplate reader. Levels of fluorescence were expressed relative to untreated controls.

### Intracellular radical oxygen species (ROS) determination

Intracellular ROS levels were determined using Cellular Reactive Oxygen Species Detection Assay Kit (Abcam; Spain). All assays were performed in duplicate, and measured on a Tecan Infinite F200 microplate reader. Levels of ROS were expressed relative to untreated controls.

### Detection of adenosine triphosphate (ATP) content

Intracellular ATP content was determined by the ATP Assay kit (Abcam; Spain). All assays were performed in duplicate, and measured on a Tecan Infinite F200 microplate reader. Levels of ATP were expressed relative to untreated controls.

### Statistical analysis

Data are expressed as the mean ± standard error of the mean (SEM). Significant differences were established using Student’s t-test or one-way analysis of variance (ANOVA), followed by Bonferroni’s post hoc test, as appropriate. Data were analysed by using the GraphPad Instat programme (GraphPad Prism 6.01 Software Inc., USA). Differences were considered statistically significant at P-value < 0.05.

## Data Availability

All relevant data are included in this published article.

## Abbreviations

HF: Heart failure
Bnp: Brain natriuretic peptide
Irps: Iron regulatory proteins
Fth1: Ferritin heavy chain 1
Ftl1: Ferritin light chain 1
Hamp: Hepcidin antimicrobial peptide
Tfr1: Transferrin receptor 1
Myh7: Myosin heavy chain 7
Dmt1: Divalent metal transporter 1
AngII: Angiotensin II
Nor: Norepinephrine
ID: Iron deficiency
RNA: Ribonucleic acid
ROS: Radical oxygen species
Fpn1: Ferroportin 1
Ftmt: Mitochondrial ferritin
Mfrn: Mitoferrin
ATP: Adenosin triphosphate
